# DNA-based species detection capabilities using laser transmission spectroscopy

**DOI:** 10.1098/rsif.2012.0637

**Published:** 2013-01-06

**Authors:** A. R. Mahon, M. A. Barnes, F. Li, S. P. Egan, C. E. Tanner, S. T. Ruggiero, J. L. Feder, D. M. Lodge

**Affiliations:** 1 Department of Biology, Institute for Great Lakes Research, Central Michigan University, Mount Pleasant, MI 48859, USA; 2 Department of Biological Sciences, University of Notre Dame, Notre Dame, IN 46556, USA; 3 Department of Physics, University of Notre Dame, Notre Dame, IN 46556, USA; 4 Advanced Diagnostics and Therapeutics, University of Notre Dame, Notre Dame, IN 46556, USA; 5 Environmental Change Initiative, University of Notre Dame, Notre Dame, IN 46556, USA

**Keywords:** DNA detection, *Dreissena*, polymerase chain reaction, laser transmission spectroscopy, ballast, invasive species

## Abstract

Early detection of invasive species is critical for effective biocontrol to mitigate potential ecological and economic damage. Laser transmission spectroscopy (LTS) is a powerful solution offering real-time, DNA-based species detection in the field. LTS can measure the size, shape and number of nanoparticles in a solution and was used here to detect size shifts resulting from hybridization of the polymerase chain reaction product to nanoparticles functionalized with species-specific oligonucleotide probes or with the species-specific oligonucleotide probes alone. We carried out a series of DNA detection experiments using the invasive freshwater quagga mussel (*Dreissena bugensis*) to evaluate the capability of the LTS platform for invasive species detection. Specifically, we tested LTS sensitivity to (i) DNA concentrations of a single target species, (ii) the presence of a target species within a mixed sample of other closely related species, (iii) species-specific functionalized nanoparticles versus species-specific oligonucleotide probes alone, and (iv) amplified DNA fragments versus unamplified genomic DNA. We demonstrate that LTS is a highly sensitive technique for rapid target species detection, with detection limits in the picomolar range, capable of successful identification in multispecies samples containing target and non-target species DNA. These results indicate that the LTS DNA detection platform will be useful for field application of target species. Additionally, we find that LTS detection is effective with species-specific oligonucleotide tags alone or when they are attached to polystyrene nanobeads and with both amplified and unamplified DNA, indicating that the technique may also have versatility for broader applications.

## 1. Introduction

Invasive species have had dramatic negative effects on freshwater and marine ecosystems, adversely impacting both biodiversity and commerce [1–7]. Economic damage caused by invasive species has been estimated at approximately $120 billion annually for the USA (both terrestrial and aquatic systems [8,9]). In the Laurentian Great Lakes alone, over 180 species of organisms have been introduced [10,11], primarily via ships' ballast, and have caused extensive and costly damage to the region. In the Great Lakes, dreissenid mussels cause over $150 million in damage annually by clogging water intake pipes in power plants, municipal water supplies and industrial facilities [12]. These and similar introductions in freshwater and marine ecosystems elsewhere have generated a need for rapid and inexpensive field-based detection technologies to identify harmful species in water samples from ballast water, ports or other at-risk areas before establishment or spread [13–16].

Research on detecting rare species has increasingly identified genetic tools as holding great promise, with a particular focus on analysis of environmental samples [17–21]. A number of techniques have been developed with varying success for the detection of aquatic organisms on site in the field. Species identification now routinely relies on forensic DNA evidence [17]. Previous studies have described novel platforms for early detection, including the use of fluorescence [22] and nanotube chips [23,24]. Although these methods have demonstrated higher specificity and increased speed in laboratory screenings of micro-organisms and plankton relative to traditional methods of hand sorting and morphologically based microscopy identification, they have high costs, low throughput, lengthy detection times (e.g. chip fabrication time and sample screening can take 2–6 h [22–24]), and dependence on technical expertise for platform preparation and operation (e.g. fluorescence microscope training [22]). More efficient methods are warranted because field-based detection and monitoring of invasive species in ships' ballast require rapid analysis and quick decisions [7,16,23].

**Figure 1.**
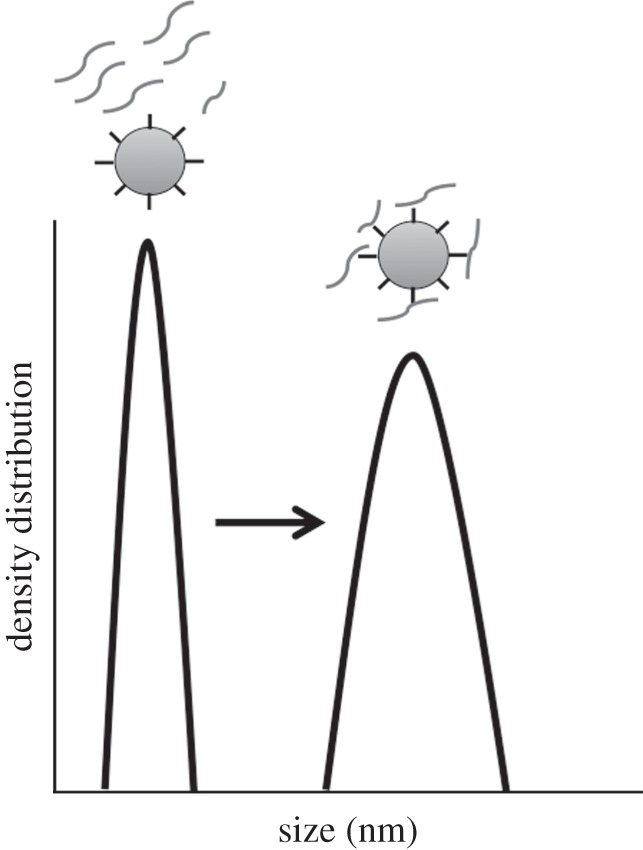
Schematic of the LTS output displaying the peak size shift resulting from target species DNA binding to species-specific oligonucleotide-tagged polystyrene beads (grey circles). See Li *et al*. [25,26] for technical details of the instrumentation and experimental protocols.

Laser transmission spectroscopy (LTS) is one method that can potentially provide rapid, cost-effective and user-friendly detection of invasive species. LTS is a quantitative detection platform for rapidly measuring the size, shape and number of nanoparticles in a solution [25,26]. The LTS platform measures wavelength-dependent light transmittance through a sample containing nanoparticles in suspension. The transmission of light through the sample cell containing particles plus suspension fluid is recorded along with that of a similar cell containing only the suspension fluid. The data are analysed and inverted by a computer algorithm that outputs particle size distribution and abundance [25]. In this procedure, double-stranded amplified DNA from a polymerase chain reaction (PCR) is briefly (2 min) denatured and subsequently incubated with tagged nanobeads. The target organism's DNA bound to species-specific DNA sequences (tags) and non-target species DNA, lacking the appropriate genetic sequence, does not bind to the tagged nanobeads [26]. When binding occurs, the increase in particle size can be detected (figure 1) to a resolution of 3 nm for mixtures, where 1 nm is roughly equivalent to 0.3–1 DNA base pair [26–28]. On the LTS platform, differentially sized molecules produce different peak profiles, depending on their size and concentration in solution. Thus, by creating species-specific oligonucleotide tags that attach only to DNA of targeted individuals or groups, the resulting LTS peak is diagnostic for the detection of an intended target. Because of this biding specificity, LTS is capable of distinguishing closely related species that differ by as few as 7 bp in a 32 bp species-specific gene region [26].

### 1.1. Rationale

Several critical questions remain unanswered concerning the efficacy of LTS for application in field settings. Here, we use experiments to evaluate DNA-based LTS screening to address the following three questions: (i) What are the concentration limits for detection using the LTS platform? (ii) How is detection affected by the presence of non-target DNA (i.e. DNA from other species)? (iii) Can LTS-based detection occur without the use of nanobeads (i.e. with only the DNA and species-specific tags present)? and (iv) Can LTS-based detection occur in the presence of unamplified genomic DNA, removing the requirement for PCR amplification? This last step, PCR-free DNA detection is critical to advancing DNA detection technologies. Thus, to move from initial studies that described LTS protocols for DNA screening [26] to practical applications using LTS as a detection platform, we used the invasive quagga mussel (*Dreissena bugensis*) as a study organism to determine whether LTS can be deployed for field-based detection of invasive species. Initial successes of the LTS platform with differentiating between closely related, congeneric species in pure water led to the question of assay sensitivity for target species, particularly at low concentrations. Additionally, determining whether LTS works in mixed samples will move the research towards more real-world conditions where genetic materials from both target and non-target organisms are present in a sample. Finally, determining whether DNA amplification (i.e. PCR) is a necessary step for successful detection on the LTS platform will demonstrate whether the techniques described here are feasible for rapid analyses while ships are under transport. We addressed these objectives by completing mixed sample tests with PCR amplified DNA and with genomic DNA, in two different experiments, one using species-specific oligonucleotide-tagged nanobeads, and one using only species-specific oligonucleotide tags.

## 2. Material and methods

### 2.1. Sample preparation: DNA extraction and amplification

Genomic DNA from target (quagga mussel) and non-target background species (zebra mussel; golden mussel; Chinese mitten crab; water flea) was obtained using a Qiagen DNEasy Blood and Tissue DNA extraction kit (Qiagen Inc.) following the manufacturer's recommended protocols. Quantification of DNA from target and non-target species (either genomic extractions or subsequent purified PCR products (see below)) was performed using a Qubit Fluorometric DNA Quantification platform (Invitrogen, Inc.). For mixed sample testing, equal concentrations of each target species were combined into a single sample for screening.

An approximately 600 bp fragment of the cytochrome *c* oxidase subunit I (COI) gene was amplified using universal primers [29] and 25 μl PCR reactions consisting of 0.75 U Taq Polymerase and 10X PCR buffer (5-Prime, Inc.), 2.5 mM Mg(OAc)_2_, 10 nmol of each dNTP, DNA template, primers and water to 25 μl. The PCR cycling programme included an initial incubation at 94°C for 1 min and 30 cycles of 94°C for 30 s, 48°C for 45 s and 72°C for 1 min. This was followed by a final extension at 72°C for 8 min. PCR products from target and from all non-target species were then purified using the Qiagen Qiaquick Gel Extraction kit (Qiagen Inc.), following an initial screening on a 1 per cent agarose gel stained with ethidium bromide.

### 2.2. Testing the limits of detection for the laser transmission spectroscopy platform under optimal conditions

To examine the ability of the LTS platform to detect rare genetic materials under optimal conditions (i.e. purified samples containing only genetic material from the target species of interest), we conducted a series of dilution trials that included a broad range of DNA concentrations to elucidate lower detection limits for the quagga mussel (table 1). We prepared a series of dilutions of purified PCR product from five quagga mussel individuals (*n* = 5). The constructed dilution curves for the five individuals contained a dilution series of 1 to 2.5 × 10^−5^ ng μl^−1^, simulating DNA levels lower than what would result from extracting DNA from a single quagga mussel veliger (approx. 30 μg of total genomic DNA [22]).

**Table 1.** Experiments and results using the LTS platform for target species DNA detection. Concentrations of genetic materials (genomic DNA or PCR product) were normalized for mixed samples (experiments 2.3 and 2.4). Sample organisms included the target quagga mussel (Q), and background species (mitten crabs, MC; golden mussels, GM; *Daphnia* *magna*, DM; and zebra mussels, ZM). Background samples consisted of equal concentrations of either DNA or PCR product from species present in the sample (MC, GM, DM and ZM).

experiment	sample organism(s)	bead + tag or tag only	genetic material in sample	results
§2.2 Testing the limits of detection for the LTS platform under optimal conditions	Q	bead + tag	PCR product	positive correlation of PCR product concentration and LTS signal
§2.3 Testing LTS detection in mixed samples (tagged nanobeads, PCR product and genomic DNA tests)	Q	bead + tag	PCR product	positive detection of target organism
	Q, GM, MC, DM, ZM	bead + tag	PCR product	positive detection of target organism
	GM, MC, DM, ZM	bead + tag	PCR product	no detection of target organism
	Q	bead + tag	genomic DNA	positive detection of target organism
	Q, GM, MC, DM, ZM	bead + tag	genomic DNA	positive detection of target organism
	GM, MC, DM, ZM	bead + tag	genomic DNA	no detection of target organism
§2.4 Testing LTS detection in mixed samples (no nanobeads, PCR and genomic DNA tests)	Q	tag only	PCR product	positive detection of target organism
	Q, GM, MC, DM, ZM	tag only	PCR product	positive detection of target organism
	GM, MC, DM, ZM	tag only	PCR product	no detection of target organism
	Q	tag only	genomic DNA	positive detection of target organism
	Q, GM, MC, DM, ZM	tag only	genomic DNA	positive detection of target organism
	GM, MC, DM, ZM	tag only	genomic DNA	no detection of target organism

Samples were screened on the LTS platform using the methods of Li *et al*. [26]. Samples containing the double-stranded DNA product (genomic DNA or PCR) were denatured by heating to 95°C for 2 min and then immediately placed on ice. Genetic samples were then combined with either functionalized, oligonucleotide-tagged polystyrene beads (1.04 × 10^9^ ml^−1^) or oligonucleotide tags (100 mM) at 48°C for 1 min. All samples were prepared in a fashion where they could be analysed blindly by researchers (i.e. no bias in interpretation of results). Samples were analysed on the LTS platform, including appropriate negative controls (solutions with no DNA; see [26]). Detections on the LTS platform were measured by comparing negative control size peaks with the peaks after hybridization with either tagged nanoparticles or tags alone (>15 nm peak shifts for these experiments). Samples were screened on the LTS platform, and peak size was quantified for each dilution for each sample, providing five measurements (initial, tagged-bead control, and four dilutions) for all five individuals. Following construction of the dilution detection curves for the target quagga mussel, we also plotted a curve of all samples to visualize the relationship between concentration and the position of the LTS peak.

### 2.3. Testing laser transmission spectroscopy detection in mixed samples with tagged beads using PCR product or genomic DNA

To examine the ability of LTS to detect target species DNA in the presence of genetic material from non-target organisms (i.e. as would occur in a real ballast sample), we prepared the following series of samples: target species only, target and multiple non-target species and non-target species only (table 1). We used simulated ballast samples because obtaining actual ballast samples from ships was not possible owing to access restrictions. Each type of sample was constructed from PCR-amplified DNA or genomic DNA only for screening on the LTS platform. The mixed species tests were intended to simulate the DNA that could be obtained from a ships' ballast sample, albeit with fewer species. The prepared samples (both PCR product and genomic DNA only) were initially tested on the LTS platform using oligonucleotide-tagged nanoparticles (table 1). Because the goal of this study was to ensure that the LTS platform is capable of detecting rare organisms in mixed samples (i.e. containing non-target species), and because the threat of even a single organism in a ballast tank could facilitate an invasion, we created our simulated samples to contain similar amounts of DNA to what is found in actual mixed samples [30].

Mixtures for all tests (PCR, DNA-only) were prepared with equal concentrations of PCR product or genomic DNA from each of the five test organisms (target and four background species; table 1). Although concentrations varied between individual trials, they were kept within detection limits found in previous experiments (typically 0.2–2.0 ng μl^−1^ for each organism; see §2.2). All prepared sample trials were performed blindly between preparation and running on the LTS instrument. All samples were measured twice on the LTS platform (i.e. two replicate readings for each sample) to ensure repeatability of the measurements. Additionally for every trial (table 1), a negative control sample was processed to determine the peak produced by the species-specific tags.

### 2.4. Testing laser transmission spectroscopy detection in mixed samples with no beads using PCR product or genomic DNA

For comparison with the bead-based PCR-product tests described earlier, we tested whether we could reduce processing time (i.e. time from sample collection to results) by eliminating beads from the assay. In addition to a reduction in sample preparation time and time-to-result, a non-bead approach could potentially offer significant risk reduction. The functionalized beads are made of polystyrene and are susceptible to heat and cold damage. For example, in the DNA binding process, the beads cannot be in the solution during the DNA denature phase as the added heat will damage the beads and return inaccurate results. The beads also have a limited lifetime (on the order of weeks), after which point their ability to bind with targeted DNA is drastically reduced (Li *et al*. 2011, unpublished data). A non-bead approach could avoid these issues. Thus, we repeated the mixed sample experiments using species-specific oligonucleotide tags only (no nanobeads) for detection of PCR or genomic DNA from target species in mixed samples, as was done in the previous experiments (table 1).

## 3. Results

### 3.1. Testing the limits of detection for the laser transmission spectroscopy platform under optimal conditions

**Figure 2.**
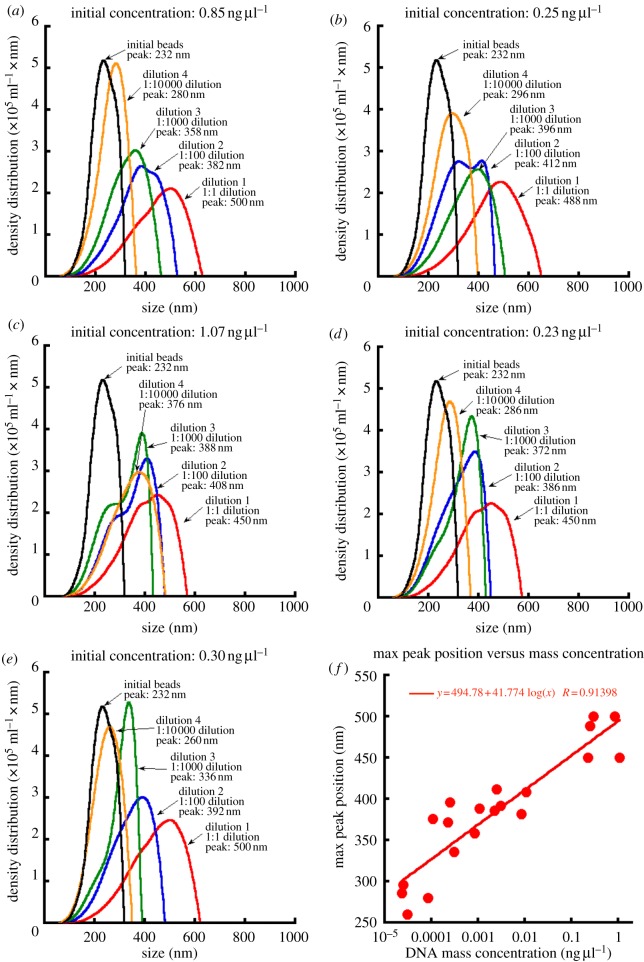
Dilution curves produced from five individual quagga mussels to elucidate limits for LTS-bead-based detection from PCR product for quagga mussel. Data from (*a*–*e*) were then used to plot the correlation of peak position to sample concentration (*f*). For each sample (*n* = 5), five dilution screenings were conducted: control (no quagga PCR product), 1 : 1 dilution, 1 : 100 dilution, 1 : 1000 dilution and 1 : 10 000 dilution.

Dilution experiments using the target quagga mussel species indicated that LTS is capable of detecting levels below the amounts of DNA found in individual target organism larvae. Tagged nanoparticles hybridized to target species DNA had larger diameters than tagged polystyrene beads alone at all concentrations, as measured by the peak of the LTS density distribution (in nm; figure 2). Tagged nanoparticles alone were measured to be 232 nm in diameter. Shifts in diameter due to ‘target DNA detection’ occurred for each of the four dilutions and all differed significantly from zero when tested by a one-sample *t*-test (mean shift in diameter ± s.e.; 1 : 1 dilution = 245.6 ± 11.5 nm, *t* = 21.4, *p* < 0.001; 1 : 100 dilution = 164.0 ± 5.97 nm, *t* = 27.5, *p* < 0.0001; 1 : 1000 dilution = 137.8 ± 10.6 nm, *t* = 12.98, *p* = 0.0002; 1 : 10 000 dilution = 67.6 ± 20.0 nm, *t* = 3.38, *p* = 0.0222). Dilution experiments with quagga mussel COI PCR product found, in general, the predicted response, where LTS density distribution peaks approached the initial tagged-bead peaks as concentration of target PCR product decreased (figure 2), suggesting fewer tag-target hybridizations at lower concentrations. Additionally, a positive linear response was found when plotting DNA concentration versus peak size across all measurements (figure 2; *y* = 41.774 log(*x*) + 494.78; *r*^2^ = 0.8354).

### 3.2. Testing laser transmission spectroscopy detection in mixed samples with tagged beads using PCR product or genomic DNA

**Figure 3.**
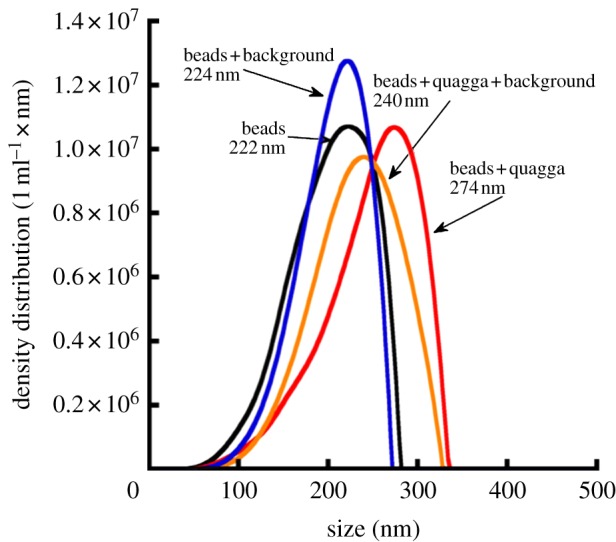
An LTS plot representing mixed sample trials using PCR product from target and non-target species (see text) and oligonucleotide-tagged nanoparticles.

By using oligonucleotide-tagged polystyrene beads, the LTS platform was able to distinguish between samples containing target and non-target species in mixed samples (figure 3). A repeated peak shift of 16–18 nm occurred in the presence of the target quagga mussel PCR product in solution with non-target background species PCR product when compared with those samples containing only tagged polystyrene beads or non-target background species PCR product (beads + background; figure 3). This is in contrast to the narrow 2.5–2.9 nm peak widths reported for repeated measurements of controls [26]. The probability that a minimum peak shift of 16 nm can be generated from the control measurements alone is less than 1/100th of a percent (*z*-test: *z*_*n* =20_ = 35.9, *p* < 0.0001). An additional peak shift of 34 nm occurred with quagga alone in solution, implying some reduced signal strength in mixed samples. Each test was run twice with 100 per cent repeatability.

### 3.3. Testing laser transmission spectroscopy detection in mixed samples with no beads using PCR product or genomic DNA

**Figure 4.**
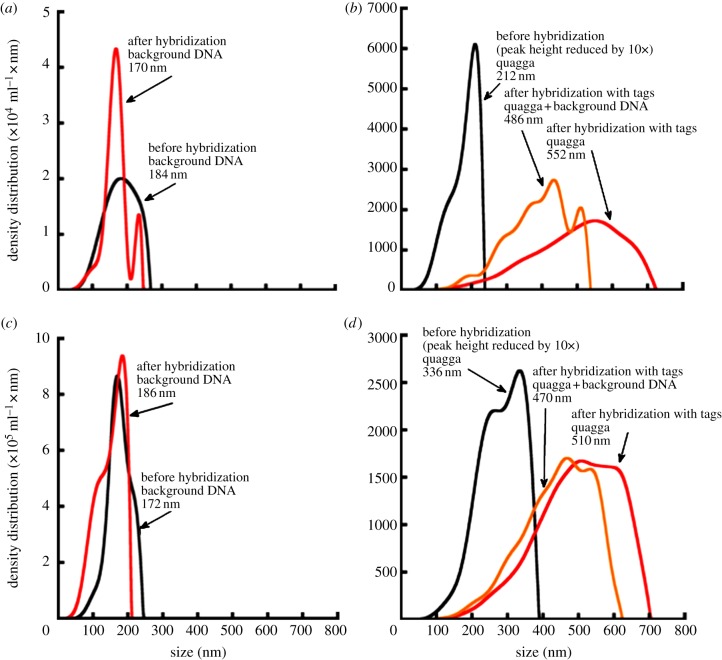
Tests examining the necessity of PCR-amplification of DNA samples, using the LTS platform. PCR and genomic DNA screenings without the use of tagged beads, i.e. only oligonucleotide primers. Both purified samples and sample + background (non-target) DNA are evaluated. (*a*) Comparison of background PCR product (black) and background PCR product plus quagga-specific tags (red). (*b*) PCR product screening: black line represents quagga PCR product with no tags; red line represents quagga PCR product only plus species-specific tags; yellow line represents quagga PCR product + background PCR product with tags. (*c*) Background DNA (black) and background DNA plus quagga-specific tags (red). (*d*) Genomic DNA screening: black line represents quagga DNA with no tags; red line represents quagga DNA only plus species-specific tags; yellow line represents quagga DNA + background DNA with quagga-specific tags.

In our experiments using oligonucleotide tags without beads, we found that for all trials (figure 4*a–d*), LTS was capable of detecting target organisms in the presence of non-target background species, whether using PCR-amplified product (figure 4*a*,*b*) or genomic DNA only (figure 4*c*,*d*). Again, each test was run twice with 100 per cent repeatability.

Negative controls (background only samples) had a maximum difference in peak position from positive controls (tag/tagged beads only) of 14 nm (figure 4*a*,*c*). All positive detections in all mixed sample screenings showed LTS peak shifts of at least 134 nm (figure 4*b*,*d*). The results for this species-specific oligonucleotide tag-only (no polystyrene beads) experiment indicated that LTS was capable of detecting target species, here quagga mussel DNA, without the use of tagged polystyrene beads.

## 4. Discussion

Real-time information about the presence and distribution of target species is necessary for shipping industry operators or government agencies to respond to the growing threat of biological invasions in aquatic environments. There is a particular need for detection tools that are not only rapid and accurate, but also easy to use in both application and interpretation of results. In this study, we demonstrate that LTS is a very sensitive technique for rapid target species detection. Using the regression model from the dilution experiments (figure 2), we can extrapolate the concentration range for potential detection to the picomolar (10^−12^) range (when *y* = 0; *x* = 1.45 × 10^−12^ ng μl DNA). In addition, we show that LTS is capable of successful identification in mixed samples containing target and non-target species DNA, a prerequisite for a method to be useful in the field with real-life samples. Additionally, LTS is also effective if species-specific oligonucleotide tags are attached to polystyrene nanobeads or free in solution, indicating that the technique may have versatility for use in other applications where nanobeads prove more cumbersome. Finally, our results suggest that it may be possible for LTS to be used ultimately to screen DNA samples collected directly from nature without the need for prior PCR amplification. This result raises the tantalizing idea that DNA-based detection can remove the amplification (i.e. PCR) step prior to detection, which represents a critical step forward for field-based DNA detection platforms.

Because of its rapid sample-to-result time, the ease of use (i.e. the platform does not require extensive technical expertise), its straightforward identification signal and its demonstrated specificity, the LTS platform holds great promise for application to industries and agencies where species detection is vital for informing management decisions. The time required to analyse a single sample is approximately 3 h (collection to final analysis), which includes samples requiring PCR amplification. If PCR can be eliminated from the procedural steps, time of analysis can be shortened to under an hour, from sample collection to final results using the LTS platform. This timing, combined with the ability to train ship crew members that lack scientific expertise to complete the processing while the ship is underway, provides advantages over other genetic methods such as PCR, quantitative PCR, restriction fragment length polymorphism, PCR-forensically informative nucleotide sequencing and other methods that require laboratories and technical experience to perform [31]. Additionally, LTS demonstrates potential to be a cost-effective and easily applied platform to identify harmful species in ships' ballast prior to ballast discharge in order to inform prevention practices. Outside the cost of the LTS instrument (not yet commercially available), sample processing and analyses costs are under $5 (USD) per sample. The LTS platform represents an important technological advance for invasive species management because the user-friendly, rapid and cost-effective sample processing could act as a trigger for additional management actions (e.g. mid-ocean ballast exchange, ballast water treatment) to increase levels of protection [32–35]. The use of existing management approaches combined with a new DNA-based technology such as LTS would be highly consistent with recent recommendations from the USEPA Scientific Advisory Board for improved ballast water-prevention practices [36].

The current study has greatly contributed to our understanding of species detection using LTS. However, several issues still remain to be resolved. The sensitivity results from the current study indicate that the LTS platform may work for many environmental DNA-based (eDNA) samples, but further tests will be necessary to determine whether our results are generalizable to other species [21]. Additionally, samples could be screened for broader taxonomic groups (e.g. genus-, family-level detection) if the appropriate oligonucleotide tags can be developed. If PCR is required, our results suggest that only a few amplification cycles may be needed for detection, but further work will be necessary to streamline workflows to half an hour or less. Additional testing is also necessary to determine whether different-sized polystyrene beads functionalized with different oligonucleotide tags can be used in the same assay to allow multiplexing of samples for detecting different species simultaneously. Quality assurance for detections of organisms that have management implications (i.e. representing new introductions) is also of great concern when implementing new technologies. Utilization of the LTS platform would have to conform to a strict, and externally auditable series of protocols that are routinely monitored, such as those described by Darling & Mahon [7], to ensure the reliability of data. Finally, we must graduate to real-time testing of samples on site in the field. This will necessitate a two-phase approach in which we first test field-collected ballast water samples spiked with known specimens for LTS detection trials conducted in the laboratory and on site followed by unadulterated samples tested in the field.

In conclusion, the current study demonstrates the potential usefulness and versatility of LTS technology for species-specific DNA detection in aqueous samples, and for particular application to ship ballast water samples. By detecting invaders early by use of the LTS platform, management and governmental groups can rapidly respond at stages where establishment can potentially be prevented or more efficiently managed [7,16].
